# Fortification of Foods with Vitamin D in India

**DOI:** 10.3390/nu6093601

**Published:** 2014-09-12

**Authors:** Ritu G, Ajay Gupta

**Affiliations:** 1 Charak Foundation, P.O. Box 3547, Cerritos, CA 90703, USA; 2 Irvine Medical Center, Division of Nephrology and Hypertension, University of California, 101 The City Dr, City Tower, Suite 400, Orange, CA 92868-3217, USA; 3 Rockwell Medical Inc., 30142 S. Wixom Road, Wixom, MI 48393, USA

**Keywords:** vitamin D, vitamin D deficiency, 25-hydroxyvitamin D, India, fortification strategies, staple foods, wheat and rice

## Abstract

Vitamin D deficiency is widely prevalent in India, despite abundant sunshine. Fortification of staple foods with vitamin D is a viable strategy to target an entire population. Vitamin D fortification programs implemented in the United States and Canada have improved the vitamin D status in these countries, but a significant proportion of the population is still vitamin D deficient. Before fortification programs are designed and implemented in India, it is necessary to study the efficacy of the American and Canadian vitamin D fortification programs and then improve upon them to suit the Indian scenario. This review explores potential strategies that could be used for the fortification of foods in the Indian context. These strategies have been proposed considering the diverse dietary practices necessitated by social, economic, cultural and religious practices and the diverse climatic conditions in India. Fortification of staple foods, such as *chapati* flour, *maida*, rice flour and rice, may be more viable strategies. Targeted fortification strategies to meet the special nutritional needs of children in India are discussed separately in a review entitled, “Fortification of foods with vitamin D in India: Strategies targeted at children”.

## 1. Introduction

Micronutrient deficiencies tend to exist where diets lack diversity and intake of animal products is minimal. Therefore, poor sectors of the world, of developing or even developed countries, may have poor diets, even in conjunction with sufficient energy intake. One of the consequences of industrialization is reduced intake of many micronutrients, because of the large dependence of the food industry on salt, sugar, vegetable fats and refined cereals, all of which are poor sources of vitamins and minerals. Individuals whose diets depend heavily on these products often do not meet the daily requirement of many micronutrients. Micronutrient malnutrition is a widespread problem throughout the world, and it has both health and economic consequences. In poor countries, this deficiency is exacerbated by systemic infections and parasitic diseases that reduce nutrient absorption and biological utilization. In the last hundred years, clinicians and nutritionists have had to shift their focus from protein sufficiency to energy sufficiency and now to micronutrient sufficiency.

The term “vitamin D” commonly refers to compounds vitamin D_3_ (cholecalciferol) or vitamin D_2_ (ergocalciferol). Vitamin D_3_ (cholecalciferol) is produced in the skin on exposure to sunlight. Vitamin D_3_ is derived from 7-dehydrocholesterol by ultraviolet irradiation of the skin. Vitamin D is photosynthesized in the skin on exposure to UVB rays. Sun exposure alone ought to suffice to attain vitamin D sufficiency. However, even in tropical countries like India, despite plentiful sunshine, vitamin D deficiency prevails in as high as 70%–100% of ostensibly healthy individuals, due to several socioeconomic and cultural constraints [1].

Fortification of widely consumed staple foods offers one of the simplest and most practical methods to combat micronutrient deficiencies for both poor and wealthy societies [1]. One of the earliest reports of food fortification dates back to 4000 BC, when the Persian physician, Melampus, added iron filings to sweet wine to strengthen the sailors’ resistance to spears and arrows and to enhance their sexual potency [2,3]. Six millenniums later, in 1833, the French chemist, Boussingault, recommended the addition of iodide to salt to prevent goiter in South America [4]. Vitamin A was added to margarine in 1920s in Denmark [5]. Vitamin D was added to milk in the United States in 1930s to help prevent rickets in children [6].

The supplementation strategy certainly has greater specificity of intervention and allows better dose adjustment. However, its disadvantages are: incurred user cost, low compliance, self-prescription and increased risk of toxicity. Fortification strategy has the advantage of universality of the intervention and greater compliance. Drawbacks of the fortification strategy are: the dose is a function of food quantity consumed; lower specificity; and varying standards legislated for each country and quality control and regulatory challenges pertaining to fortification levels with the manufacturers of fortified foods.

Fortification of staple foods with vitamin D offers a viable solution to address the vitamin D deficiency epidemic in India [1]. The Indian diet is amenable to fortification with vitamin D. India has the scientific expertise to examine and implement fortification of food with vitamin D, either commercially or at the level of the local community or in an individual home. The aim of this article is to provide seminal information to encourage, examine and implement population-based strategies for the fortification of foods with vitamin D. The aim of this review is not to design a national level vitamin D fortification program, but only to discuss the pros and cons of several challenges and possibilities that may be faced in India while designing such programs. The objectives are to: (1) discuss the efficacy of vitamin D fortification strategies in the USA and Canada; (2) discuss the technological and economic feasibility of vitamin D fortification strategies in India; and (3) review vitamin D fortification strategies most suited to the Indian scenario. Targeted fortification strategies to meet the special nutritional needs of children in India are discussed in a separate review [7].

## 2. Vitamin D Intake Required to Achieve Optimal Vitamin D Status

The issue of optimal vitamin D status is as yet unsettled. In accordance with the Endocrine Society’s Clinical Practice Guidelines, the lower serum threshold for 25(OH)D level is defined here as 75 nM or 30 ng/mL [8,9,10]. The Institute of Medicine (IOM) recommended intake (600 IU/day) for all individuals (1–70 years) does not suffice to attain sufficiency (≥30 ng/mL) [10]. The extra intake of vitamin D needed through supplements or fortified foods, in addition to general food intake patterns and endogenous production, is 1000–4000 IU/day [10]. A recommended daily allowance (RDA), by definition, must meet the need of 97.5% of the population. Conformably, ICMR’s (Indian Council of Medical Research) RDA values, for both vitamin D (400 IU/day) and calcium (600 mg/day for adults) need revision. Notably, most naturally available vitamin D-rich foods are scarce, and the dietary options are further limited for the predominantly vegetarian populace of India. Additionally, not only is milk unaffordable to socioeconomically disadvantaged people in India, it is also rarely fortified in India.

## 3. Vitamin D Fortification Strategies in the USA and Canada

### 3.1. Vitamin D Fortification Policies in the USA and Canada

In the USA, fortification of milk with vitamin D began in the 1930s [6]. Originally, milk was fortified with vitamin D by irradiating milk or by feeding the cows irradiated yeast. This technique was replaced in the 1940s by the simpler and more effective method of adding vitamin D concentrate to milk, as is practiced today. In the USA, vitamin D fortification of foods is voluntary, but it is strictly regulated pertaining to the categories of foods, the functional use and level of use, thus limiting over-fortification. Vitamin D is added to most milk sold in the United States, although it is not added to all milk products, like cheese and ice cream. Cheese and cheese products are permitted fortified foods in the USA. Some companies also add vitamin D to breakfast cereals, soy milk, rice milk and orange juice, usually along with calcium. In Canada, the law mandates fortification of milk (180 IU/250 mL), milk alternatives and margarine (530 IU/100 gm). Similar to USA, for other permitted foods, vitamin D fortification is voluntary, but the fortification level is limited. Most RTE (ready-to-eat) breakfast cereals in the United States are fortified with vitamin D, whereas few yogurts or margarines are fortified with vitamin D. Fortification of RTE breakfast cereals with vitamin D is unlawful in Canada [11]. Lists of selected foods fortified which are permitted in the USA were published in a 2013 review [11]. A normal serving of food is considered at least 40 calories, *i.e.*, 2% of a daily intake of 2000 Calories. Fortification of vitamin D in permitted foods is not to exceed 20 IU/100 Calories.

### 3.2. Efficacy of Vitamin D Food Fortification Strategies in North America

Vitamin D fortification and supplementation strategies implemented in the USA and Canada have significantly improved the vitamin D status in these nations. Notwithstanding these interventions however, according to the National Center for Health Statistics data for March, 2011, vitamin D status of the United States (2001–2006), 33% of persons aged one year and over had serum 25(OH)D values <20 ng/mL [12]. In another review, vitamin D deficiency was reported to be still widely prevalent in the USA, with about 36% of individuals at <20 ng/mL [13]. Most natural food sources with significant levels of vitamin D are non-vegetarian. The North American diet is predominantly non-vegetarian. Notwithstanding non-vegetarianism, more than 60% of the intake of vitamin D from food comes from fortified foods in the USA [14,15] and Canada [16].

The potential of vitamin D fortification strategies to improve vitamin D status was reviewed [17], and its efficacy was studied by meta-analyses [18,19]. Foods fortified with vitamin D usually contain 100 IU per serving. Consumption of vitamin D fortified foods, especially milk, increased vitamin D intake and was effective in significantly increasing 25(OH)D levels. Other foods included some cereals, juices, other dairy products and margarine. A mean individual intake of approximately 11 μg/day (440 IU/day) from fortified foods (range 120–1000 IU/day) increased 25(OH)D concentrations by 7.7 ng/mL. This corresponded to a 0.5 ng/mL increase in 25(OH)D for each 40 IU (1 μg) ingested/day [18]. A major contributor of vitamin D from fortified foods in both countries was milk, contributing 44% to total daily vitamin D intake. The next major contributor among vitamin D fortified foods was RTE cereals in the USA [20,21]. Notably, young males between 13 and 18 years [21] and, according to another study, males aged 9–18 years [22] had the highest vitamin D intakes among the age/sex categories. In both of the studies, older women had the lowest intakes [21,22]. In both countries, population mean intakes of vitamin D-rich foods and vitamin D fortified foods itself was lower and did not meet the current estimated average requirement (EAR) level of 400 IU in accordance with IOM’s serum levels of 16 ng/mL [23]. Therefore, when ≥30 ng/mL is considered as the vitamin D sufficiency level, the present population mean intake of vitamin D becomes much lower than what is required in both the USA and Canada. In other words, a higher proportion of the population would not meet the daily vitamin D requirement. Arguably, the fortification level of foods in both the USA and Canada is insufficient. Public health advisory of higher consumption of the existing foods fortified with vitamin D may be impractical. Higher levels of fortification of foods are needed to increase the percentage of individuals with 25(OH)D ≥ 20 ng/mL in the population.

### 3.3. Vitamin D Fortification Strategies in the Developing World

Implementation of fortification programs, especially in the developing world, has been lackadaisical. There may be several reasons: (1) a lack of information pertaining to micronutrient deficiency status; (2) a lack of understanding of the significance of micronutrient deficiencies and the causal burden on the healthcare system; (3) inadequate information about food consumption patterns; (4) the food industry’s concerns about consumer acceptance, costs and competitive impacts; and (5) a lack of proactive political and administrative leadership to plan, initiate, monitor and sustain mass fortification programs.

Several vitamin D fortification programs have been implemented in developing countries. These programs have been summarized previously [24]. Mostly milk, milk products and margarine were fortified with vitamin D. Often foods were fortified with vitamin D in conjunction with vitamin A [24]. However, information pertaining to the continuation and success of these programs is scant.

## 4. A Necessitated *Obiter Dictum*: Vitamin A

Vitamin A has been used to fortify foods in several other matrices in developing countries [24]. Vitamin A and vitamin D share certain properties, like fat solubility, sensitivity to light, moisture, oxidation and (to a certain extent) thermal stability. Both vitamins, being fat soluble, also have toxicity issues. During extrusion, both vitamin A and vitamin D behave similarly [25]. Thermally, vitamin D is significantly more robust than vitamin A. Several foods, like cooking oils, margarine, milk and cereal flour, have been used for vitamin A fortification [5]. These food items are also permitted and used for vitamin D fortification in the USA and Canada [11]. Additionally, sugar has been fortified with vitamin A in South America. During the 1970s, fortification of sugar with vitamin A was first implemented in Guatemala [5,26,27], followed by other Central American countries, including Costa Rica, Honduras and El Salvador [24]. Although the programs were suspended at various times since their inception due to political and other constraints, all three countries operated successful sugar-fortification programs, as reported in a 2002 review [24]. However, updated information is scant.

## 5. Fortification of Foods with Vitamin D in India: A Bespoke Strategy Is Needed

Vitamin D deficiency with its multifarious effects on health status, levies a huge burden on the healthcare system worldwide. Several advanced nations have launched nationwide fortification programs to improve vitamin D status. India must follow suit. Foods are rarely fortified with vitamin D in India. Limitations pertaining to vitamin D fortified milk, Amul™ (Anand, Gujarat, India) and Kellogg’s breakfast cereals are discussed in a previously published review [1], in Section 20. Two vitamin D fortification pilot studies in ostensibly healthy subjects were reported from India [28,29]. These are discussed in detail in a previously published review [1], in Section 18. These studies support the strategy of the fortification of foods in India for redressing malnutrition problems in India. They also suggest that to reach sufficiency, a daily intake of more than 1000 IU may be required to attain vitamin D sufficiency.

## 6. Technological Feasibility of Vitamin D Fortification of Foods in India

This section discusses various technological aspects of the fortification of foods with vitamin D in India, e.g., availability of fortificant(s), suitable vehicles for the fortificant(s) and certain situations specific to the Indian scenario.

1. Technical expertise to produce D_2_ and D_3_ is available. D_3_ is already produced and marketed in India.
2. D_2_ and D_3_ forms of vitamin D have the same effect on raising blood levels of 25-hydroxyvitamin D [30]. Equivalent efficacy of D_2_ and D_3_ pertaining to the anti-rachitic properties [31] is a great benefit, especially with vegetarians in mind. Either may be used, alone or in combination.
3. Although vitamin D is a fat-soluble vitamin, it can be added to both “fat-rich” products, such as whole milk or cheese, and to “fat-poor” foods, such as skim milk, fat free yogurt, orange juice, *etc*. Several formulations of vitamin D are available to suit all matrices [32,33]. Dry stabilized vitamin D is available and contains an antioxidant (usually tocopherol) which protects the potency of vitamin D for much longer, even in the presence of minerals.
4. The selection of suitable fortification vehicles will play an important role in India. India has a plethora of suitable and readily available fortification vehicles, which have already been tested and proven in other parts of the world. Some examples are: milk and milk products, *chapati* flour, rice, rice flour, soy milk, fruit juices, breakfast cereals, sugar, salt, *etc*.
  4.1 The fortification vehicle must be a widely and regularly consumed staple food or a more processed, commercially available and affordable food, without seasonal variation in its consumption.
  4.2 The fortification vehicle must allow a nutrient premix to be added relatively easily using low-cost technology and in such a way so as to ensure an even distribution, within different batches of the product.
  4.3 The bioavailability of vitamin D in fortified foods, such as milk, milk powder, cheese [34], bread [35,36], yogurt [37], orange juice [30,38,39], *etc*., is good [19]. The bioavailability of vitamin D from fortified foods fortified with vitamin D in oil-based and non-oily formulations is acceptable. However, there is no clear evidence available yet as to which expedients and fillers used along with the active principle give better results [33].
  4.4 Vitamin D fortification of foods has a minimal sensory effect [40,41,42].
5. Stability of vitamin D: vitamin D is susceptible to UV light, air (oxygen) and acids. Some studies also observed degradation of vitamin D by hydrolyzed proteins [43]. Vitamin D is very stable at sub-zero temperatures and 4–8 °C. It is also stable at 25 °C for weeks. Thermal transformation of vitamin D_3_ may occur between 110 and 170 °C, with an inverse relationship between temperature and time of heating [44]. However, vitamin D is generally considered as a relatively robust vitamin, which is stable during cooking up to 200 °C. The percent recovery of vitamin D_3_ in fatty fish, like salmon, when fried in vegetable oil was only 50%, while the recovery from baked fish was almost 100% [45]. Stability of vitamin D_3_ during cheese processing and aging of cheddar cheese and low fat cheese was 91% and 55%, respectively [46]. Vitamin D_3_ exhibits >90% stability in fortified foods, including high-temperature short-time-processed 2% milk, UHT (ultra-high temperature processing; heating milk for 1–2 s, at >135 °C)—processed 2% fat chocolate milk and low fat strawberry yogurt [40] and orange juice after 30 days of storage at 4 °C [39]. Evidently, vitamin D withstands the extrusion methods of the processed-food industry fairly well [25].
6. Methods of measuring vitamin D in foods are available [47,48] and will be important in regulating and enforcing the levels of vitamin D added to foods.

## 7. Economic Feasibility of Vitamin D Fortification of Foods in India

The economic feasibility of food fortification would be much better than the supplementation strategy in India, as discussed in a previously published review [1], in Section 18. A noteworthy publication entitled, “Indian social safety net programs as platforms for introducing wheat flour fortification: A case study of Gujarat, India” analyzed the cost of fortifying wheat flour in the state of Gujarat, India, with vitamin A, iron, zinc, *etc*. The results were encouraging [49].

### Drawbacks

1. Mandatory food fortification (Box 1) is politically contentious. The perception of the uninformed and uneducated masses is generally that their food supply is being tampered with. The populace would need to be educated about the need for such measures.
2. Stringent and aggressive regulations are needed to ensure quality control and steady supply of fortified foods.
3. Fortification may also be of limited use for those with low energy intake, such as infants and young children. Targeted fortification strategy is used for their benefit.

## 8. Voluntary *versus* Mandatory Fortification Strategies in India

Three basic types of fortification strategies may be implemented in India (Box 1). Strategies in India would need to overcome conventional barriers to use adequate fortification levels in staple foods. A mandatory food fortification policy may be unacceptable in the initial stages, due to misconceptions and perceptions of the uninformed and uneducated masses. The option of voluntary fortification without a significant difference in the price of the product would certainly eliminate political reticence regarding fortification. Consumers respond well to voluntary fortification of food. The USA provides a good example, where it is difficult to find milk unfortified with vitamin D on the store shelves. As policymakers, manufactures and consumers are educated about the necessity and impact of vitamin D fortification, a strategic shift from voluntary to mandatory fortification may be possible.

**Box 1. Types of food fortification strategies.**

Classified by who they are aimed at and how they are regulated.

1. Mandatory or mass fortification of staple foods, such as milk, cereal flour, *etc.*, is mandated and regulated by the government. It is implemented when the majority of the population has a serious public health issue of being or becoming deficient in specific micronutrients, as in the case of mandatory fortification of milk and milk products and margarine with vitamin D in Canada to address the problem of vitamin D deficiency.
2. Targeted fortification is the practice of adding sufficient amounts of micronutrients to provide large proportions of the daily needs through foods designed for specific population subgroups, such as complementary foods for infants and foods for institutional programs, such as those aimed at pre-school and school-aged children. These foods may provide a large proportion of the daily micronutrient requirements for specific target groups. A separate review addresses this strategy for children in India [7].
3. Market-driven or voluntary fortification is a prerogative of a food manufacturer who voluntarily adds one or more micronutrients to processed foods with the purpose of adding value to their products, thereby increasing marketability and sales. The micronutrient(s) added and the levels of fortification are regulated by the government.

## 9. Determination of Adequate Level of Vitamin D Fortification in India

The levels of vitamin D fortification needed in foods may vary depending on the gap between the epidemiological realities of vitamin D status of the target population and well-defined goals. Goals need to be well defined not only for the general population, but also for specific sub-groups of the population that may have special needs, e.g., infants, young children, pregnant women, lactating mothers and senior citizens.

WHO guidelines for setting fortification levels in foods are available [32]. There are some publications using other algorithms, too. However, a detailed discussion on these methods is beyond the purview of this review. Resolution of vitamin D deficiency has two phases: (1) increasing 25(OH)D levels to more than 30 ng/mL; and (2) maintenance of serum 25(OH)D between 30 and 100 ng/mL. The objective of the fortification strategy is not to address recommended intakes of vitamin D in totality. Supplementation will always be needed for some subgroups of the population to reach the minimally desired 25(OH)D, *i.e*., 30 ng/mL. That said, the fortification level of foods needs to be established based on per capita consumption and the size of the vitamin D intake gap in India. Vitamin D is associated with toxicity issues. A desirable range is 30–100 ng/mL. The margin of safety for vitamin D plays a substantial role in determining permissible levels of fortification. The ratio of the targeted intake (RDA) to the upper limit (UL) for the nutrient (presently 4000 IU/day based on the IOM) determines the permissible excess intake beyond the RDA. The serum 25(OH)D levels of people whose bare skin is exposed to abundant sunshine, as in some African aborigines, commonly range from 40 ng/mL (100 nM) to 90 ng/mL (225 nM) [50,51]. Therefore, the intake of vitamin D that can bring about such levels of 25(OH)D may be considered physiological and safe.

Determination of vitamin D fortification levels requires the following data: (1) A study of the quality of the diet of the target population and an estimation of the gaps in micronutrient intakes. Even when dietary information is scant, objectives can be initially defined based on the knowledge that consumption of dairy and other animal products is minimal in developing countries and to a lesser extent in India, due to widely practiced vegetarianism. (2) Average sun exposure is insufficient to attain vitamin D sufficiency levels, for most Indians, due to socioreligious constraints [1]. (3) The current vitamin D status in India [1]. Once a fortification program is established, continual monitoring and evaluation efforts could be used to obtain better dietary information and, more importantly, efficacy of vitamin D fortification levels used in the Indian populace. Subsequently, this information may be used to adjust the fortification levels. It is very likely that in a country like India, with diverse dietary practices, the determination of fortification levels may require data from several regions and ethnicities of the country, to arrive at algorithm(s) to suit the Indian scenario.

## 10. How to Achieve ≥ 30 ng/mL 25(OH)D Serum Levels by Fortified Foods Alone in India

The IOM’s 2011 Dietary Reference Intakes (DRIs) [52] are consistent with the estimated average requirement (EAR) of 400 IU and the recommended dietary allowance (RDA) of 600 to 800 IU for those aged ≥1 year. The intake set as EARs and RDAs assume minimal sun exposure. There is no consensus on how to calculate fortification levels. In the USA, mean individual intake of approximately 11 μg/day (440 IU/day) from fortified foods (range, 120–1000 IU/day) increased 25(OH)D concentrations by 7.7 ng/mL, corresponding to approximately a 0.5 ng/mL increase in 25(OH)D for each 40 IU (1 μg) ingested [18].

The daily intake needed in India may be estimated by using the following example (see Section 11). This example assumes that dietary intake is uniform for all individuals, which is clearly not so in the general population. Assuming that the Indian population has 25(OH)D levels of 10 ng/mL and our goal is to attain ≥30 ng/mL, concordant with the Endocrine Society’s Clinical Practice Guidelines, we would need to make up for a deficiency of 20 ng/mL. Serum 25(OH)D levels increase by 0.5 ng/mL for each 40 IU/day intake [18]. Therefore, we would need 1600 IU/day or 40 μg/day intake for Indians.

## 11. Why Would We Need to Aim for 40 μg (1600 IU/day) in India?

- It raises serum 25(OH)D level by at least 20 ng/mL
- An intake of 11 μg (440 IU/day) is insufficient, even in the USA.
- The current intake of 440 IU/day in the USA does prevent rickets, but about 36% individuals in the USA are still <20 ng/mL [13].
- It would put many Indians over 30 ng/mL and may be effective for not only vitamin D’s skeletal, but also extraskeletal benefits.
- In fact, a safe intake of 50 μg (2000 IU) may serve the Indians well, bearing in mind their vegetarian diets, the high phytate content in the Indian diet, dark skin, conservative clothing and already poor nutritional status. A level of 2000 IU is also well below the safe upper limit (UL) of 4000 IU [8].
- The North American diet, which is predominantly rich in meats, dairy produce and eggs, contributes 5 μg (200 IU); therefore, the Indian diet, which is predominantly vegetarian and sparing in dairy produce, must contribute a significantly lesser amount.
- Using higher levels of vitamin D fortification in staple foods may be worth the while in the long run, convenient and affordable, especially when the tatterdemalion state of the already heavily burdened and poorly funded Indian healthcare system is taken into consideration.

## 12. Fortification of Staple Foods

Several foods may be fortified with vitamin D in India, e.g., milk, yogurt, infant formulas, *ghee*, soy milk, sugar, *etc*. These are discussed in some more detail in a previously published review [1], in Section 19. However, widely consumed and affordable staple food items, such as *chapati* flour, *maida* (all-purpose wheat flour), rice and rice flour, may be some of the most suitable vehicles for fortification strategies in the Indian scenario. No country currently adds vitamin D to cereal staples. However, it is often added to complementary foods based on cereal and/or legumes targeted for children. Some staple foods are discussed in more detail later.

Staple foods are widely consumed by all ages and socioeconomic groups with little variation in day-to-day consumption and without seasonal variation. To a large extent, staple foods are processed at central facilities. This would be facilitative to the process of fortification and its regulation. As food prices go up, consumers often stop buying as many meats, fruits and vegetables, but there is little or no change in the consumption of staple foods. Fortification of staple foods, such as *chapati* flour, *maida*, rice or rice flour, puts more vitamins and minerals in staple foods, which people continue to purchase and consume during economic downturns too. Fortification of staple foods offers a clear advantage: all of the foods, whether cooked in individual homes or processed at the industrial level, which use these staples also get fortified as a consequence (Table 1). Perhaps staple foods should be fortified with vitamin D and calcium together to maximally leverage the benefits of both micronutrients. A meta-analysis showed the need for calcium and vitamin D in conjunction to reduce the risk of hip fractures [53]. Wheat and rice are the two most consumed cereals in India. Derivatives of wheat flour (including bread and other bakery products) and rice flour (*idli*, *dosa* and *uthapam*; these are discussed later) closely follow the consumption of wheat flour and rice depending on the region(s) of India. Wheat flour, *maida*, rice and rice flour may also be considered good food vehicles for mandatory fortification, given their high consumption relative to other foods in the country. Cereal flours are versatile vehicles for fortificants. Several micronutrients may be added together without causing any perceptible sensory change. The low moisture content of flours is ideal for the stability of fortificants (such as vitamin D), which are susceptible to moisture. Thus, cereal flour fortified with vitamin D may have a high shelf life compared to other staple foods, such as milk. The feasibility of vitamin D fortification of some staple foods is discussed in the following section.

The concept of the fortification of staple foods with regard to India or Indians is not novel. In 1976, consumption of fortified *chapati* flour (wheat flour), by subjects of Indian origin in Glasgow, was shown to significantly raise serum 25(OH)D levels. Vitamin D fortification of *chapati* flour was proposed in Britain to benefit the immigrants of Indian origin [54]. In 1978, a study of vitamin D intake and dietary habits of infants of Indian origin in the U.K., aged six and 18 months, concluded that to meet the nutritional needs of children of Indian origin, aged one years and above, vitamin D fortified *chapati* flour would be most suited [55]. In 1996, a review on osteoporosis in India discussed the U.K. department of Health and Human Services’ rejection of compulsory fortification of *chapati* flour for immigrants of Indian origin. This review suggested strategies for mass prevention of calcium and vitamin D deficiency by fortification of foods with these nutrients in India [56]. In 2003, vitamin D fortification of milk and milk products in India was suggested [57]. A 2010 review proposed the development of a national vitamin D food fortification program for *chapati* flour/wheat flour [58]. The aforementioned citations are only those that were retrieved from PubMed, by using the following search words: vitamin D, food fortification and India. Factually, most Indian researchers who have published their work pertaining to vitamin D deficiency have decried the lack of vitamin D fortified foods in India. In this review, we have attempted to discuss possible strategies and their pros and cons when possible. We cannot stress enough the diverse dietary, cultural, ethnic and climactic conditions in India, and thus, the need for the bespoke strategies that are possibly most suitable to specific regions of India.

### 12.1. Milk

Milk, including dry and evaporated milk, is generally the preferred vehicle to fortify with vitamin D all over the world. Milk is rarely fortified with vitamin D in India. India is the largest producer of dairy milk in the world. Nevertheless, milk is unaffordable to the socioeconomically underprivileged, due to higher demand and inadequate supply. Milk supplied in India is not homogenized. There is significant stratification upon storage in unhomogenized milk, with a higher lipid concentration found in the upper layers. Since vitamin D is fat soluble, to achieve a uniform distribution of added vitamin D throughout milk, homogenization of milk will be required. Homogenization of milk will significantly reduce variability in vitamin D concentration from lot to lot, which is likely to happen in unhomogenized milk. However, the practice of homogenization will further increase the cost of milk in India. A major concern in India is the rampant dilution and/or adulteration of milk and milk products. Another factor that may be a deterrent for consumption of milk and/or milk products, especially in India, is the high prevalence of lactose intolerance [59,60,61,62].

**Table 1:** Foods that may be suitable for fortification with vitamin D in India.

Food Category	Staple Food Fortified	Widely Consumed Related Products Fortified as a Consequence
**Foods that May Be Considered for Mandatory Fortification**		
Milk and milk products	Milk	All grades of milk, curd (plain yogurt) and cheese.
Cereal flour and related products	Whole wheat flour	*Chapati*
	*Maida* (all-purpose wheat flour)	*Naan* (flatbread made of leavened dough), instant (or not) noodles, pasta, *seviyan* (vermicelli), pizzas, bakery products, e.g., bread, rusk, burger buns, biscuits/cookies, cakes, *etc*.
	Corn flour	Corn *chapati*
	Corn starch	All products (extruded products included) using corn starch.
	Rice	All rice dishes
	Rice flour	*Idlis*, *etc.,* and also all extruded products using rice flour.
**Foods that May Be Considered for Voluntary or Market Oriented Fortification**		
Sugar	Table sugar or *boora* (powdered sugar)	All already cooked beverages, snacks and dishes sweetened with table sugar.
Salt	Salt	All already cooked foods to which salt is added after cooking.
Oils and fats used as spreads or to spike already cooked food	Butter	All already cooked foods spiked with butter.
	*Ghee* (clarified butter)	All already cooked foods spiked with *ghee*.
*Dalia* (porridge)		
Semolina (*suji*)		
Canned fruit juices		

Foods that require shallow or deep frying have not been mentioned. When foods are fried, vitamin D in the food comes out into the cooking medium and is thermally degraded [45]; vitamin D is fairly robust and is stable during cooking. The term “already cooked” foods is used here only as a precautionary stance so as to not overstate vitamin D’s thermal stability.

### 12.2. Wheat Flour

*Chapati* (unleavened flatbread) is widely consumed in India. Fortification of *chapati* flour (whole wheat flour) with vitamin D is a very viable fortification vehicle. Flour of several kinds of cereals may be used to make *chapatis*, e.g., wheat, corn, millet (*bajra*), sorghum/milo (*jawar*), barley (*jav*), *etc*. The most commonly used cereal is wheat flour. *Chapatis* are consumed by all ages, socioeconomic backgrounds and by the rural and urban populations, too. The quantity of flour consumed varies little from day to day, thus it is a suitable vehicle for fortification. Indian children start eating *chapatis* at around 12 months of age and then gradually eat about 20 g of flour a day by the time they are 2–3 years old. Adults in the north and central India generally consume 150–200 gm of flour daily. India is the world’s second largest producer of wheat, with an annual production of about 90 million tons [63]. In 14 of India’s 25 major states (which together have more than 60% of India’s population), wheat is the main staple food. These states would be the obvious initial targets for introducing wheat flour fortification.

The technological feasibility and bioavailability of vitamin D in fortified wheat flour was tested in *chapatis* [54] bread [35,36,64] with encouraging results. Notably, bread fortified with 5000 IU of vitamin D_3_ per daily serving consumed by sun-deprived nursing home residents in Romania (latitude: 47°N) demonstrated both the efficacy and safety of wheat flour fortification. Fortification of bread resulted in higher serum 25(OH)D concentrations (>30 ng/mL) and significantly increased bone density [64].

### 12.3. Maida (All-Purpose Wheat Flour)

*Maida* is highly refined wheat flour. Fortification of *maida* will have a significant contribution in the improvement of the vitamin D status of the general population. When flour is fortified at the mill, it causes all of the many products made from that flour to also be fortified. Households purchase only small quantities of *maida*, primarily for making snacks and sweets. Most of the *maida* produced in India is consumed by bulk consumers, *i.e*., thousands of bakeries, hotels, restaurants and extruded food industries that produce hundreds of other products that will be consequently fortified, as well. Pertaining to baked products, most of the vitamin losses occur during baking, which is the most common process that all wheat flour products go through. Although baking temperatures are high (over 200 °C), the temperature inside the product is significantly lower, so the thermal degradation of vitamin D would be within an acceptable range.

### 12.4. Rice

Rice is the most widely consumed staple food in southern and eastern India. In other parts of the country, also, the consumption of rice follows closely that of wheat flour’s. Some fortification technologies and characteristics of the fortified rice are mentioned below (Box 2). To cater to the needs of all socioeconomic strata, all low-end and high-end rice varieties would need to be fortified eventually. Milled white rice in India generally has a powdery residue sticking to the surface of the kernels. Starch, glucose, talc powder or other coatings may be used to improve the appearance of the grains (“polished rice”, though this term may also refer to white rice in general). Additionally, there may be some dirt, too, more so in the cheaper varieties of rice sold in India. Rinsing with water 3–4 times is required before cooking. Removal of powdered residue also prevents the cooked rice kernels from sticking to each other. Hence, rinsing is a ritual strictly adhered to by all Indian cooks. This rinsing, however, will wash away any fortificant dusted or coated on the rice kernels. Therefore, use of simulated kernels packed with vitamin D may be a better choice in India, until rice milling technologies and quality control measures in India can assuredly supply rice which is residue and debris free, too.

**Box 2. Fortification technologies and characteristics of the fortified rice [65].**

**Hot Extrusion**Dough made of rice flour, a fortificant mix and water passes through an extruder and cuts it into grain-like structures that resemble rice kernels. This process involves relatively high temperatures (70–110 °C). It results in fully or partially pre-cooked simulated rice kernels that have similar appearance (sheen and transparency) as regular rice kernels.**Cold Extrusion**Rice-shaped simulated kernels are produced by passing dough made of rice four, a fortificant mix and water through a simple pasta press. There is no thermal energy input other than the heat generated during the process itself. Temperature is maintained below 70 °C. Grains produced are uncooked, opaque and easier to differentiate from regular rice kernels.**Coating**Combines the fortificant mix with ingredients, such as waxes and gums. The mixture is sprayed on the rice on the surface of grain kernels in several layers to form the rice-premix and then is blended with polished rice.**Dusting**Used in the USA, this involves dusting the polished rice grains with the powder form of the micronutrient premix. The fortificant(s) sticks to the grain surface due to electrostatic forces. It is the most economical method. The label on the packaging states “to retain the micronutrients, do not rinse/wash rice before cooking”.The first three processes produce a rice-premix that is blended with retail rice (polished rice packaged at rice mills). The fourth applies a micronutrient-premix directly to rice.

### 12.5. Rice Flour

Fortification of rice flour may prove to be advantageous, especially in southern India, where rice flour is used to prepare *idlis*, *dosas* and *uthapam*: *idlis—*steamed rice cakes, usually 2–3 inches in diameter*—*are made by steaming a fermented batter consisting of black lentils (de-husked) and rice; *dosa—*a crepe made from fermented batter of rice and black lentils (de-husked); *uthapam—*a pancake made from fermented batter of rice and black lentils (de-husked). Relatively, rice flour used for *idli* is coarser than that used for *dosa*. These savory dishes are staple foods in the south, and are consumed as a snack, breakfast item or a regular meal. These dishes are also very popular in the rest of the country, but are not consumed to the same extent. The advantage of rice flour is that vitamin D powder formulation may be added to the flour at the mill itself. The disadvantage is that most people in southern India still prefer to wash rice to their satisfaction and then to soak the rice and black lentils and grind the batter to their own specifications. Several brands of premixes for *idli* and *dosa*/*uthapam* are available in stores in most metropolises already. Due to the change in the pace of life, especially in the big cities, the ease of preparation from such premixes is attractive. The purchase of rice flour from the market will assuredly become more popular and preferred, as consumers are assured about the good manufacturing practices (GMP) adhered to by the manufacturers, and as the consumers are educated about the benefits of vitamin D fortification.

### 12.6. Cooking Oils and Spreads like Butter and Ghee (Clarified Butter)

Vitamin D is fat soluble; thus fats, margarine and cooking oils are attractive fortification vehicles for vitamin D all over the world [11,24,66]. Fortification of margarine with vitamin D is mandatory in Canada and optional in the USA [11]. Cooking oils are not fortified in the USA and Canada [67]. In relative terms, vitamin D is more robust than many other vitamins. It is generally believed to be stable during cooking. However, there are publications reporting significant losses, too [45]. Some details are included in the section pertaining to technological feasibility. Most cooking fats and oils have low specific heat and high smoke points (>180 °C) [68,69]. Thus, most cooking oils would be unsuitable for vitamin D fortification. There is an ever rising demand for refined cooking oils in the market. The consumers prefer the neutral taste of these refined oils. The process of refining renders the cooking medium a lighter texture and significantly raises the smoke points, thus making the oils a better choice for frying foods. Due to their high smoke points, cooking oils may not be suitable vehicles for vitamin D fortification. The consumers would also need to be educated about the fact that any food fortified with vitamin D, if fried, will result in the loss of most of the vitamin D in the food. Vitamin D is fat soluble; it will come out in the cooking oil and will be thermally degraded. In a study with a vitamin D-rich dietary source, like wild salmon, frying resulted in degradation of 50% of the vitamin D content in the fish, while baking did not result in a significant loss of vitamin D [45]. To reduce the loss of fat-soluble vitamins, cooking with very little oil is advisable, *i.e*., baking or broiling (oil-free) instead of frying (shallow or deep).

Lessons from past experiences must guide our future strategies. A few decades ago, hydrogenated vegetable oil/*vanaspati* (with more than 50% “trans fats”) was fortified with vitamin C. Incidentally, vitamin C is the most heat labile vitamin with significant losses above 40°C. Suffice it to say, fortification with vitamin C was eventually discontinued due to inherent technological limitations. Some states in India have implemented programs to supply edible oils fortified with vitamin A and D through public distribution systems. However, information regarding the efficacy of these programs is unavailable. Notably, cooking oil manufacturers generally comply with fortification strategies with alacrity, perhaps to leverage the marketability of their “spuriously wholesome” products. Cooking oils fortified with vitamins A, D and E are available in India, but most people are not aware of them and their claimed benefits.

Butter and *ghee* (clarified butter) may be fortified, but with clear instructions to be added only to already cooked food. There must be clear instructions on the label that vitamin D will be destroyed if these fats are used for frying foods. Other edible fats and oils should not be used for vitamin D fortification. In spite of the inherent limitations of the efficacy of vitamin D fortification of cooking oils, if fortification of fats and oils is permitted, it must necessitate warnings emblazoned on the packaging that overconsumption of these products may exacerbate other health issues related to metabolic syndrome. Metabolic syndrome is already plaguing most of the middle and upper classes in India.

### 12.7. Sugar

In 2010–2011, India was the second largest producer of sugarcane in the world with 342 million tons [63] and 24.3 million tons of sugar produced. India occasionally exports sugar. Factually, India imports sugar often to meet its ever growing domestic need. Around 65% of domestic demand comes from industrial and bulk consumers. Individual consumers contribute to the remaining 35% [70]. The food industry’s demand has been strong in recent years, due to rising demand for sweets, cakes and pastries, as a result of changing food habits. Sugar available in India has much larger crystals (about 1 cubic mm). Therefore, the emulsified vitamin D powder formulation added may gradually settle at the bottom of the packaging upon storage. *Boora* (powdered or confectioner’s sugar), which is generally used to make Indian sweets, may be a better vehicle for fortification. No publication was available pertaining to the fortification of sugar with vitamin D. Several South American countries have sugar fortified with vitamin A—another fat soluble vitamin [5,24,26,27]. However, updated information on the status and efficacy of these programs is lacking.

## 13. Factors Contributing to the Success and Sustenance of Fortification Programs in India

1. Political and administration’s will and support should be available from the development stage of the fortification program and, equally important, sustained.
2. To gain the committed and sustained support of the food industry, the following steps will be necessary:
  2.1 The government will need to provide necessary information to the producers of fortified foods. The food industry would require updated information and dependable evidence to be able to make business decisions in favor of continued fortification and adherence to standards defined by the government. The government would need to provide support in terms of technical expertise pertaining to the production of fortified food items and the availability of standardized vitamin D formulation(s). The industry will also need information regarding types of vitamin D formulations suitable for specific fortification vehicles; levels of fortification for all different food types; and the marketing potential of the fortified items. Data pertaining to the impact of fortification on micronutrient intake and nutrition status would be most helpful.
  2.2 The government will need to offer attractive incentives and subsidies to the food industry participating directly (manufacturing) or indirectly (promoting) in the fortification program.
  2.3 Participation of all community-based, small-scale, medium-scale and large-scale producers of fortified foods would be needed.
  2.4 Involvement of the food industry by encouraging private enterprises operating at the national and local level would be required.
3. Education: To maximize a program’s effectiveness, the perceptions and attitude of the populace towards nutrition and the need for vitamin D sufficiency may need to be modulated. Winning active consumer’s interest, cooperation and participation will depend on the awareness pertaining to the benefits and necessity of vitamin D sufficiency. Vitamin D deficiency is the world’s most under-diagnosed and under-treated disease. There is great need for awareness of its implications among physicians and the general public at large.
4. Consumers’ cooperation may be won if the additional cost of fortification for the consumers is minimal and they can be assured of a steady supply of a well-regulated quality of fortified foods.
5. Provision for affordable and widely accessible testing facilities for serum vitamin D levels at a subsidized cost may help in two ways. Consumers may determine for themselves, if they so choose, that: (1) their vitamin D status is suboptimal; and (b) the fortified foods are indeed boosting their vitamin D levels.
6. Monitoring and evaluation is a critical factor in the sustainability of fortification programs. In a country like India, rife with problems of food adulteration and corruption at all levels, perhaps affordable and easily accessible facilities to test levels of vitamin D fortification in staple foods would be extremely advantages. These facilities could be made available at minimal cost, to all distributors, retailers and consumers. This measure ought to ensure quality control of fortified foods at multiple levels and benefit the fortification program in the long run.
7. Complete transparency of government’s goals, efforts and achievements through continual updates *via* mass media and social activists would be helpful. In the long run, this transparency will benefit all of the stakeholders of the fortification program—the food industry, consumers, government, administration and also the heavily burdened and poorly funded Indian healthcare system.
8. Effective legislation to ensure good quality and regulated vitamin D fortified foods at minimal cost to the end consumer will definitely be required. In this regard, legislature should allow expedited punitive actions for the saboteurs and unethical profiteers. That said, both the administration and legislature should very importantly be facilitative and not be restrictive or punitive to those who are striving towards attaining better bone and mineral health in India.
9. Serum vitamin D levels and health outcomes, especially bone-related, would need to be continually monitored to assess the efficacy of the fortification programs. The data would guide the fine-tuning of the fortification levels in India and also would impart tremendous credibility to the fortification program.
10. Lastly, but imperatively, the government’s policies on fortification should support and expedite regional, national and also international initiatives to promote and enable “healthy eating habits” with respect to both macro- and micro-nutrients.

## 14. A Novel Strategy: Fortification of Creams and Body Lotions with Vitamin D for General Use

Skin is the largest organ of the body with a great absorption capacity. The VDR (vitamin D receptor) is abundantly expressed in skin. Keratinocyte, the major cell of the epidermis, not only expresses VDR, but also expresses enzymes (25-hydroxylase and 1 alpha-hydroxylase) to synthesize the active form of vitamin D-1,25(OH)_2_D [71]. 1,25(OH)_2_D induces keratinocyte differentiation. Topical vitamin D formulations, specifically the active hormone, 1,25(OH)_2_D, and its analogs, are used to treat psoriasis [72]. Additionally, sebocytes are 1,25(OH)_2_D-responsive target cells, and vitamin D may prove to be effective in the treatment of acne [73,74]. There are several creams and lotions, available on the internet, which contain vitamin D_3_ (cholecalciferol).

Body lotions and creams fortified with vitamin D may prove to be a very fashionable way to keep the skin glowing from within and without. Indians are very conscious about skin tone and complexion. The addition of SPF (sun protection factor) in such cosmetic formulations, if feasible, would be a bonus, *i.e*., a single-step application for moisturizing the skin, vitamin D supplementation and reduced threat of tanning. Such a product will win loyal consumers, even from rural India. Though the concept of vitamin D supplementation through body lotions and creams is very attractive, continued research is warranted to ascertain its merit.

## 15. A “Do-It-Yourself” Project

While waiting for fortification strategies to be developed and implemented nationwide, individuals could fortify their own foods. Information useful to fortify foods at home is as follows:

- Sachets of vitamin D (cholecalciferol) could be added to various food stuffs: wheat flour, rice flour, powdered sugar (*boora*), *ghee* (clarified butter), salt, *ghee* (only used to spike already cooked food), fruit juices, *etc*.
- Sachets of 60,000 IU for INR 25 are available at the chemist’s shop (drug store/pharmacy) in the Indian market. One sachet is sufficient for a family of four adolescent/adult members for 15 days at 1000 IU per person per day.
- The forms of vitamin D that can be used for fortification are: (1) liquid D_3 _ in oil, 60,000 IU, in transparent capsules may be used to fortify *ghee* or oil spreads; and (2) sachets of the powder form of D_3_, 60,000 IU, may be used to fortify all other foods.
- The cost per person per day: The retail price of the vitamin D 60,000 IU sachet is INR 25. Thus, it will cost INR 1.67 for four people (at 1000 IU each day per person). For one person per day, the cost will be INR 0.42.
- Safety concern: 60,000 IU of Vitamin D_3_ is okay to be taken as a bolus dose by one person. This amount of vitamin D_3_ or D_2_ is not toxic. In a study, an intake of 50,000 IUs of vitamin D_2_ every two weeks for six years was reported to be safe [75]. The intake of three packets of 60,000 IU every day for several weeks may lead to toxicity. However, is there any food or drink that would not be toxic if consumed at 100-times the daily amount for several weeks? The answer is: NO
- Two examples of fortification at home are as follows:
  (a) The average wheat flour consumption per person per day is about 200 gm. Mix contents of 1 sachet to 2.1 kg of wheat flour thoroughly and store in a cool, dark and dry place. This premix will be sufficient for four persons for 15 days. Add one cup (1 cup = 140 g) to flour before kneading the dough once every day for a family of four. This will provide about 1000 IU of vitamin D per person per day.
  (b) Add the contents of one sachet of vitamin D in 300 mL of condensed milk, mix thoroughly and store in an amber glass jar in the refrigerator. Consume 5 mL or one teaspoon every day after a meal or add once per day to milk, brewed tea or coffee. This will provide about 1000 IU of vitamin D every day.

This do-it-yourself project, especially with home-fortified cereal flour, may prove to be a very valuable strategy while we wait for the governments’ interventions for the following reasons: (1) better compliance by all of the members of the family partaking of home cooked meals; (2) steady and sustained intake of vitamin D by all members of the family; and (3) no discrimination against girls, women and senior citizens within the household.

## 16. Conclusions

The epidemic of vitamin D deficiency in India and its health consequences call for a population-based approach, such as food fortification rather than prescribing vitamin D supplements. Fortification doses in India would need to be adjusted to suit the vitamin D status and dietary patterns of Indians. Considering the diversity of foods and cultures, a large variety of foods will need to be fortified. We propose the addition of vitamin D to wheat flour, *maida*, rice and rice flour in India, to increase mean daily dietary intakes of vitamin D. A significant overall improvement in bone health and general health can be anticipated with minimal risk at a modest cost, because of the ability to capitalize on existing technology and distribution systems. A detailed analysis of the economic feasibility of mandatory vitamin D fortification in India, of staple foods, such as milk, milk products, wheat flour, *maida*, rice and rice flour, is warranted.

## References

[1] G R., Gupta A. (2014). Vitamin D deficiency in india: Prevalence, causalities and interventions. Nutrients.

[2] Richardson D.P. (1990). Food fortification. Proc. Nutr. Soc..

[3] Panda A.K., Mishra S., Mohapatra S.K. (2011). Iron in ayurvedic medicine. J. Adv. Dev. Res..

[4] Cowgill G.R. (1964). Jean baptiste boussingault—A biographical sketch (2 February 1802–11 May 1887). J. Nutr..

[5] Dary O., Mora J.O., International Vitamin A Consultative Group (2002). Food fortification to reduce vitamin a deficiency: International vitamin a consultative group recommendations. J. Nutr..

[6] Rajakumar K., Greenspan S.L., Thomas S.B., Holick M.F. (2007). Solar ultraviolet radiation and vitamin D: A historical perspective. Am. J. Public Health.

[7] G R., Gupta A. (2014). Fortification of foods with vitamin D in India: Strategies targeted at children. J. Am. Coll. Nutr..

[8] Holick M.F., Binkley N.C., Bischoff-Ferrari H.A., Gordon C.M., Hanley D.A., Heaney R.P., Murad M.H., Weaver C.M., Endocrine S. (2011). Evaluation, treatment, and prevention of vitamin D deficiency: An endocrine society clinical practice guideline. J. Clin. Endocrinol. Metab..

[9] Holick M.F., Binkley N.C., Bischoff-Ferrari H.A., Gordon C.M., Hanley D.A., Heaney R.P., Murad M.H., Weaver C.M. (2012). Guidelines for preventing and treating vitamin D deficiency and insufficiency revisited. J. Clin. Endocrinol. Metab..

[10] Heaney R.P., Holick M.F. (2011). Why the IOM recommendations for vitamin D are deficient. J. Bone Miner Res..

[11] Calvo M.S., Whiting S.J. (2013). Survey of current vitamin D food fortification practices in the United States and Canada. J. Steroid Biochem. Mol. Biol..

[12] Looker A.C., Johnson C.L., Lacher D.A., Pfeiffer C.M., Schleicher R.L., Sempos C.T. (2011). Vitamin D status: United States, 2001–2006. NCHS Data Brief..

[13] Hossein-Nezhad A., Holick M.F. (2013). Vitamin D for health: A global perspective. Mayo Clin. Proc..

[14] Whiting S.J., Calvo M.S. (2006). Overview of the proceedings from experimental biology 2005 symposium: Optimizing vitamin D intake for populations with special needs: Barriers to effective food fortification and supplementation. J. Nutr..

[15] Fulgoni V.L., Keast D.R., Bailey R.L., Dwyer J. (2011). Foods, fortificants, and supplements: Where do Americans get their nutrients?. J. Nutr..

[16] Langlois K., Greene-Finestone L., Little J., Hidiroglou N., Whiting S. (2010). Vitamin D status of Canadians as measured in the 2007 to 2009 Canadian health measures survey. Health Rep..

[17] O’Mahony L., Stepien M., Gibney M.J., Nugent A.P., Brennan L. (2011). The potential role of vitamin D enhanced foods in improving vitamin D status. Nutrients.

[18] Black L.J., Seamans K.M., Cashman K.D., Kiely M. (2012). An updated systematic review and meta-analysis of the efficacy of vitamin D food fortification. J. Nutr..

[19] O’Donnell S., Cranney A., Horsley T., Weiler H.A., Atkinson S.A., Hanley D.A., Ooi D.S., Ward L., Barrowman N., Fang M. (2008). Efficacy of food fortification on serum 25-hydroxyvitamin D concentrations: Systematic review. Am. J. Clin. Nutr..

[20] Keast D.R., Fulgoni V.L., Nicklas T.A., O’Neil C.E. (2013). Food sources of energy and nutrients among children in the United States: National health and nutrition examination survey 2003–2006. Nutrients.

[21] Hill K.M., Jonnalagadda S.S., Albertson A.M., Joshi N.A., Weaver C.M. (2012). Top food sources contributing to vitamin D intake and the association of ready-to-eat cereal and breakfast consumption habits to vitamin D intake in Canadians and United States Americans. J. Food Sci..

[22] Vatanparast H., Calvo M.S., Green T.J., Whiting S.J. (2010). Despite mandatory fortification of staple foods, vitamin D intakes of canadian children and adults are inadequate. J. Steroid. Biochem. Mol. Biol..

[23] Ross A.C., Manson J.E., Abrams S.A., Aloia J.F., Brannon P.M., Clinton S.K., Durazo-Arvizu R.A., Gallagher J.C., Gallo R.L., Jones G. (2011). The 2011 dietary reference intakes for calcium and vitamin D: What dietetics practitioners need to know. J. Am. Diet Assoc..

[24] Darnton-Hill I., Darnton-Hill I., Nalubola R. (2002). Fortification strategies to meet micronutrient needs: Successes and failures. Proc. Nutr. Soc..

[25] Riaz M.N., Asif M., Ali R. (2009). Stability of vitamins during extrusion. Crit. Rev. Food Sci. Nutr..

[26] Arroyave G., Mejia L.A., Aguilar J.R. (1981). The effect of vitamin a fortification of sugar on the serum vitamin A levels of preschool guatemalan children: A longitudinal evaluation. Am. J. Clin. Nutr..

[27] Fiedler J.L., Helleranta M. (2010). Recommendations for improving guatemala’s food fortification program based on household income and expenditure survey (hies) data. Food Nutr. Bull.

[28] Ekbote V.H., Khadilkar A.V., Chiplonkar S.A., Hanumante N.M., Khadilkar V.V., Mughal M.Z. (2011). A pilot randomized controlled trial of oral calcium and vitamin D supplementation using fortified laddoos in underprivileged Indian toddlers. Eur. J. Clin. Nutr..

[29] Khadgawat R., Marwaha R.K., Garg M.K., Ramot R., Oberoi A.K., Sreenivas V., Gahlot M., Mehan N., Mathur P., Gupta N. (2013). Impact of vitamin D fortified milk supplementation on vitamin D status of healthy school children aged 10–14 years. Osteoporos Int..

[30] Biancuzzo R.M., Young A., Bibuld D., Cai M.H., Winter M.R., Klein E.K., Ameri A., Reitz R., Salameh W., Chen T.C. (2010). Fortification of orange juice with vitamin D_2_ or vitamin D_3_ is as effective as an oral supplement in maintaining vitamin D status in adults. Am. J. Clin. Nutr..

[31] Thacher T.D., Fischer P.R., Obadofin M.O., Levine M.A., Singh R.J., Pettifor J.M. (2010). Comparison of metabolism of vitamins D_2_ and D_3_ in children with nutritional rickets. J. Bone Miner. Res..

[32] Allen L.H., Benoist B., Dary O., Hurrell R., World Health Organization, Food and Agricultural Organization of the United Nations (2006). Guidelines on Food Fortification with Micronutrients.

[33] Grossmann R.E., Tangpricha V. (2010). Evaluation of vehicle substances on vitamin D bioavailability: A systematic review. Mol. Nutr. Food. Res..

[34] Wagner D., Sidhom G., Whiting S.J., Rousseau D., Vieth R. (2008). The bioavailability of vitamin D from fortified cheeses and supplements is equivalent in adults. J. Nutr..

[35] Natri A.M., Salo P., Vikstedt T., Palssa A., Huttunen M., Karkkainen M.U., Salovaara H., Piironen V., Jakobsen J., Lamberg-Allardt C.J. (2006). Bread fortified with cholecalciferol increases the serum 25-hydroxyvitamin D concentration in women as effectively as a cholecalciferol supplement. J. Nutr..

[36] Madsen K.H., Rasmussen L.B., Andersen R., Molgaard C., Jakobsen J., Bjerrum P.J., Andersen E.W., Mejborn H., Tetens I. (2013). Randomized controlled trial of the effects of Vitamin D-fortified milk and bread on serum 25-hydroxy vitamin D concentrations in families in denmark during winter: The vitmad study. Am. J. Clin. Nutr..

[37] Bonjour J.P., Benoit V., Payen F., Kraenzlin M. (2013). Consumption of yogurts fortified in vitamin D and calcium reduces serum parathyroid hormone and markers of bone resorption: A double-blind randomized controlled trial in institutionalized elderly women. J. Clin. Endocrinol. Metab..

[38] Biancuzzo R.M., Clarke N., Reitz R.E., Travison T.G., Holick M.F. (2013). Serum concentrations of 1,25-dihydroxyvitamin D2 and 1,25-dihydroxyvitamin D3 in response to vitamin D2 and vitamin D3 supplementation. J. Clin. Endocrinol. Metab..

[39] Tangpricha V., Koutkia P., Rieke S.M., Chen T.C., Perez A.A., Holick M.F. (2003). Fortification of orange juice with vitamin D: A novel approach for enhancing vitamin D nutritional health. Am. J. Clin. Nutr..

[40] Hanson A.L., Metzger L.E. (2010). Evaluation of increased vitamin D fortification in high-temperature, short-time-processed 2% milk, uht-processed 2% fat chocolate milk, and low-fat strawberry yogurt. J. Dairy Sci..

[41] Upreti P., Mistry V.V., Warthesen J.J. (2002). Estimation and fortification of vitamin D3 in pasteurized process cheese. J. Dairy Sci..

[42] Ganesan B., Brothersen C., McMahon D.J. (2011). Fortification of cheddar cheese with vitamin D does not alter cheese flavor perception. J. Dairy Sci..

[43] Gregory J.F., Richardson T., Finley J.W. (1985). Chemical changes of vitamins during food processing. Chemical Changes in Food during Processing Basic Symposium Series.

[44] Pelc B., Marshall D.H. (1978). Thermal transformation of cholecalciferol between 100–170 degrees C. Steroids.

[45] Lu Z., Chen T.C., Zhang A., Persons K.S., Kohn N., Berkowitz R., Martinello S., Holick M.F. (2007). An evaluation of the vitamin D3 content in fish: Is the vitamin D content adequate to satisfy the dietary requirement for vitamin D?. J. Steroid. Biochem. Mol. Biol..

[46] Wagner D., Rousseau D., Sidhom G., Pouliot M., Audet P., Vieth R. (2008). Vitamin D3 fortification, quantification, and long-term stability in cheddar and low-fat cheeses. J. Agric. Food Chem..

[47] Byrdwell W.C., Devries J., Exler J., Harnly J.M., Holden J.M., Holick M.F., Hollis B.W., Horst R.L., Lada M., Lemar L.E. (2008). Analyzing vitamin D in foods and supplements: Methodologic challenges. Am. J. Clin. Nutr..

[48] Holden J.M., Lemar L.E. (2008). Assessing vitamin D contents in foods and supplements: Challenges and needs. Am. J. Clin. Nutr..

[49] Fiedler J.L., Babu S., Smitz M.F., Lividini K., Bermudez O. (2012). Indian social safety net programs as platforms for introducing wheat flour fortification: A case study of gujarat, India. Food Nutr. Bull..

[50] Luxwolda M.F., Kuipers R.S., Kema I.P., Dijck-Brouwer D.A., Muskiet F.A. (2012). Traditionally living populations in east Africa have a mean serum 25-hydroxyvitamin D concentration of 115 nmol/L. Br. J. Nutr..

[51] Luxwolda M.F., Kuipers R.S., Kema I.P., van der Veer E., Dijck-Brouwer D.A., Muskiet F.A. (2013). Vitamin D status indicators in indigenous populations in east Africa. Eur. J. Nutr..

[52] Ross A.C., Manson J.E., Abrams S.A., Aloia J.F., Brannon P.M., Clinton S.K., Durazo-Arvizu R.A., Gallagher J.C., Gallo R.L., Jones G. (2011). The 2011 report on dietary reference intakes for calcium and vitamin D from the institute of medicine: What clinicians need to know. J. Clin. Endocrinol. Metab..

[53] Boonen S., Lips P., Bouillon R., Bischoff-Ferrari H.A., Vanderschueren D., Haentjens P. (2007). Need for additional calcium to reduce the risk of hip fracture with vitamin D supplementation: Evidence from a comparative metaanalysis of randomized controlled trials. J. Clin. Endocrinol. Metab..

[54] Pietrek J., Preece M.A., Windo J., O’Riordan J.L., Dunnigan M.G., McIntosh W.B., Ford J.A. (1976). Prevention of vitamin-D deficiency in Asians. Lancet.

[55] Singleton N., Tucker S.M. (1978). Vitamin D status of Asian infants. Br. Med. J..

[56] Gupta A. (1996). Osteoporosis in India—The nutritional hypothesis. Natl. Med. J. India.

[57] Joshi S.R., Joshi S.S. (2003). Vitamin D deficiency and tuberculosis: Need for vitamin D food fortification. J. Assoc. Physicians India.

[58] Babu U.S., Calvo M.S. (2010). Modern India and the vitamin D dilemma: Evidence for the need of a national food fortification program. Mol. Nutr. Food Res..

[59] Tandon R.K., Joshi Y.K., Singh D.S., Narendranathan M., Balakrishnan V., Lal K. (1981). Lactose intolerance in north and south Indians. Am. J. Clin. Nutr..

[60] Babu J., Kumar S., Babu P., Prasad J.H., Ghoshal U.C. (2010). Frequency of lactose malabsorption among healthy southern and northern Indian populations by genetic analysis and lactose hydrogen breath and tolerance tests. Am. J. Clin. Nutr..

[61] Gallego Romero I., Basu Mallick C., Liebert A., Crivellaro F., Chaubey G., Itan Y., Metspalu M., Eaaswarkhanth M., Pitchappan R., Villems R. (2012). Herders of indian and european cattle share their predominant allele for lactase persistence. Mol. Biol. Evol..

[62] Hollox E.J., Poulter M., Zvarik M., Ferak V., Krause A., Jenkins T., Saha N., Kozlov A.I., Swallow D.M. (2001). Lactase haplotype diversity in the old world. Am. J. Hum. Genet.

[63] Agricultural Production—Foodgrains, Ministry of Agriculture, Government of India. http://rbidocs.rbi.org.in/rdocs/Publications/PDFs/017T_BST130913.pdf.

[64] Mocanu V., Stitt P.A., Costan A.R., Voroniuc O., Zbranca E., Luca V., Vieth R. (2009). Long-term effects of giving nursing home residents bread fortified with 125 μg (5000 IU) vitamin D_3_ per daily serving. Am. J. Clin. Nutr..

[65] Alavi S., Bugusu B., Cramer G., Dary O., Lee T., Martin L., McEntire J., Wailes E. Rice Fortification in Developing Countries: A Critical Review of the Technical and Economic Feasibility. http://pdf.usaid.gov/pdf_docs/PNACD279.pdf.

[66] Yang Z., Laillou A., Smith G., Schofield D., Moench-Pfanner R. (2013). A review of vitamin D fortification: Implications for nutrition programming in southeast Asia. Food Nutr. Bull..

[67] Calvo M.S., Whiting S.J., Barton C.N. (2004). Vitamin D fortification in the United States and Canada: Current status and data needs. Am. J. Clin. Nutr..

[68] Smoke Point. http://en.wikipedia.org/wiki/Smoke_point.

[69] Cooking Oil Smoke Points. http://www.goodeatsfanpage.com/collectedinfo/oilsmokepoints.htm.

[70] Jha D.K. First Annual Sugar Consumption Rise after Three Years. http://www.business-standard.com/article/markets/first-annual-sugar-consumption-rise-after-three-years-111060300102_1.html.

[71] Bikle D.D. (2012). Vitamin D and the skin: Physiology and pathophysiology. Rev. Endocr. Metab. Disord..

[72] Kurian A., Barankin B. (2011). Current effective topical therapies in the management of psoriasis. Skin Ther. Lett..

[73] Lee W.J., Cha H.W., Sohn M.Y., Lee S.J., Kim do W. (2012). Vitamin D increases expression of cathelicidin in cultured sebocytes. Arch. Dermatol. Res..

[74] Zouboulis C.C., Baron J.M., Bohm M., Kippenberger S., Kurzen H., Reichrath J., Thielitz A. (2008). Frontiers in sebaceous gland biology and pathology. Exp. Dermatol..

[75] Pietras S.M., Obayan B.K., Cai M.H., Holick M.F. (2009). Vitamin D2 treatment for Vitamin D deficiency and insufficiency for up to 6 years. Arch. Intern Med..

